# Dose-dependent protective effects of p-coumaric acid on testicular ischemia/reperfusion injury in a mouse model of testicular torsion

**DOI:** 10.1016/j.bbrep.2026.102760

**Published:** 2026-08-23

**Authors:** Khashayar Khorsand, Alireza Najafpour, Mohammadreza Valilou, Ali Soleimanzadeh

**Affiliations:** a Department of Clinical Sciences, Faculty of Veterinary Medicine, Islamic Azad University, Ur.C., Urmia, Iran; b Department of Veterinary Pathobiology, Tabriz Medical Sciences, Islamic Azad University, Tabriz, Iran; c Department of Theriogenology, Faculty of Veterinary Medicine, Urmia University, Urmia, Iran

**Keywords:** p-coumaric acid, Testicular torsion, Oxidative stress, Spermatogenesis, Dietary phenolic acid, Antioxidant, Male fertility

## Abstract

Testicular torsion triggers ischemia/reperfusion (I/R) injury that drives oxidative stress, germ cell apoptosis, and long-term impairment of spermatogenesis and fertility. This study investigated dose-dependent protective effects of p-coumaric acid (p-CA), a phenolic molecule with well-documented antioxidant, anti-inflammatory, and anti-apoptotic properties, in a mouse model of testicular torsion. Forty adult male mice underwent 2 h of 720° unilateral testicular torsion, followed by detorsion. Animals were randomly assigned to sham, torsion/detorsion (T/D), and three T/D + p-CA groups. Thirty minutes before detorsion, the mice received intraperitoneal p-CA at 50, 100, or 200 mg/kg. Sperm quality (concentration, motility, and kinematics) was evaluated in the right testis and epididymis after a 30-day recovery period. Fertility outcomes, testicular histology, oxidative stress markers, and molecular indicators of apoptosis were also assessed. The T/D procedure markedly diminished sperm quality, lowered antioxidant capacity (TAC, SOD, GPx), reduced hormone levels, elevated malondialdehyde (MDA), and increased apoptotic markers (Bax, caspase-3). Administration of p-CA attenuated these changes in a dose-dependent manner. Doses of 100 and 200 mg/kg most effectively improved sperm parameters, enhanced antioxidant defenses, preserved seminiferous tubule architecture, and suppressed apoptosis. Only moderate improvement was observed with the 50 mg/kg dose. Collectively, the results suggest that p-CA exerts robust protective effects against testicular ischemia/reperfusion injury, preserving spermatogenesis and fertility. To our knowledge, this is the first study to characterize the dose-dependent protective effects of p-CA in a mouse model of testicular torsion/detorsion, integrating detailed sperm kinematics, molecular apoptotic profiling, and actual fertility outcomes within a single experimental framework. These findings extend existing knowledge on phenolic antioxidants and highlight the translational potential of p-CA as a perioperative adjunct in the clinical management of testicular torsion.

## Highlights

• p-Coumaric acid protects against testicular torsion-induced I/R injury in mice.
• Dose-dependently improves sperm quality, motility, and fertility rates.
• Enhances antioxidants (TAC, SOD, GPx) while reducing MDA and apoptosis.
• Restores histological integrity and hormonal balance at 100-200 mg/kg.

## 1 Introduction

Testicular torsion, a medical emergency, occurs when the spermatic cord twists. Delayed diagnosis and intervention can result in testicular atrophy and necrosis. Testicular torsion is the primary cause of testicular loss in neonates, children, and adolescent males. The severity and duration of torsion are the critical determinants of the extent of testicular injury [1]. The affected testicle can be preserved with medical intervention within 6 h of pain onset, achieving a survival rate of 90–100%. Salvage rates diminish sharply thereafter. Immediate surgical detorsion is essential to restore blood supply to the ischemic testicle. Nonetheless, reperfusion of ischemic tissue often exacerbates injury [2].

Reperfusion following detorsion paradoxically amplifies tissue damage [3,4]. The excessive generation of pro-inflammatory cytokines during the ischemia and reperfusion phases. Consequently, leukocytes, particularly neutrophils, migrate to the testicular tissue, generating deleterious reactive oxygen species (ROS) that damage testicular cells [[5], [6], [7]]. This cascade promotes lipid peroxidation, excessive release of cellular calcium, and oxidative stress, whereby peroxides and free radicals damage proteins, lipids, DNA, and other cellular components [8].

Antioxidants have demonstrated considerable potential in reducing I/R-induced damage across multiple organs, including the testis, primarily by scavenging free radicals [9]. p-Coumaric acid (4-hydroxycinnamic acid), a prevalent phenolic compound present in fruits, vegetables, and grains, possesses well-documented antioxidant, anti-inflammatory, and anti-apoptotic properties [10,11]. Previous studies have demonstrated the efficacy of p-CA across several I/R models; for instance, p-CA has shown protective effects in cerebral I/R injury through attenuation of oxidative stress and inflammation [12,13].

However, data on its efficacy in testicular torsion remain limited: most prior antioxidant studies in this field have relied on rat models, and none have combined dose optimization, long-term fertility outcomes, detailed sperm kinematics, and molecular apoptotic profiling of p-CA within a single mouse model. The present study therefore aimed to address these gaps by evaluating the dose-dependent protective effects of intraperitoneal p-CA (50, 100, and 200 mg/kg), administered 30 min before detorsion, in a mouse model of severe testicular torsion/detorsion. We assessed impacts on sperm quality (concentration, motility, kinematics), histological parameters, expression of key apoptotic regulators (Bcl-2, Bax, caspase-3), oxidative stress markers, and fertility indices. By optimizing dosing and delivery while incorporating comprehensive reproductive endpoints, this work seeks to strengthen the evidence for p-CA as a clinically translatable adjunct in the management of testicular I/R injury.

## 2 Methods

### 2.1 Chemicals

p-Coumaric acid (Sigma-Aldrich, St. Louis, MO, USA; catalogue no. C9008; ≥98% purity, HPLC) and other requisite chemical compounds were supplied by esteemed suppliers, including Merck (Darmstadt, Germany) and Sigma (St. Louis, MO, USA).

### 2.2 Animals

Forty adult male mice (20–25 g, 6–8 weeks old) were obtained from the Animal Resource Center of Medical Sciences. Animals were housed under controlled conditions (12-h light/dark cycle, 50 ± 10% humidity, 20–22°C) with ad libitum access to standard laboratory diet and tap water. Following one week of acclimatization, mice were randomly assigned to experimental groups.

### 2.3 Experimental protocol

Mice were randomly allocated to five groups (n = 8 each).

1. Group 1: Sham-operated control (no torsion).
2. Group 2: Torsion/detorsion (T/D).
3. Groups 3–5: T/D + p-CA (50, 100, or 200 mg/kg) [14].

p-CA or vehicle was administered intraperitoneally 30 min prior to detorsion [15].

### 2.4 Surgical procedure

Under anesthesia (ketamine 80 mg/kg and xylazine 10 mg/kg, i.p.), the scrotal area was shaved and disinfected. The right testis (unilateral torsion model) was exteriorized through a midline incision and rotated 720° clockwise for 2 h. After 2 h, detorsion was performed and the incision closed. Thirty days post-detorsion, animals were euthanized under deep surgical anesthesia via an intraperitoneal overdose of ketamine (80 mg/kg) and xylazine (10 mg/kg), consistent with the anesthetic protocol used for the surgical procedure, for tissue and blood collection [16].

### 2.5 Testosterone concentrations and hematological sampling

On day 30, blood samples were collected, centrifuged (2000 g, 10 min, 4°C), and plasma stored at −20°C. Testosterone concentrations were measured using a commercial ELISA kit (Monobind Inc., USA) according to the manufacturer's instructions [16].

### 2.6 Collection of epididymal spermatozoa

The right cauda epididymis was minced in 1 mL human tubal fluid (HTF) medium and incubated (37°C, 5% CO_2_, 30 min) [[17], [18], [19]].

### 2.7 Sperm analysis

#### 2.7.1 Epididymal sperm count

A Neubauer hemocytometer was employed to enumerate the sperm after they were made nonviable and diluted 1:5 in distilled water. The quantity of sperm per gram of tissue was determined by weighing epididymal fragments [20].

#### 2.7.2 Motility of epididymal spermatozoa

Motility was assessed via computer-assisted semen analysis (CASA) at room temperature (Table 1). Parameters included total motility (%), progressive motility (%), amplitude of lateral head displacement (ALH, μm/s), curvilinear velocity (VCL, μm/s), straight-line velocity (VSL, μm/s), average path velocity (VAP, μm/s), straightness (STR, %), linearity (LIN, %), and beat-cross frequency (BCF, Hz). At least 500 spermatozoa were analyzed in five fields using phase-contrast microscopy [21].

**Table 1:** Computer-assisted semen analysis (CASA) parameter settings used in the study.

Parameter	Setting
**Frame rate**	60-Hz
**Number of frames**	30
**Duration of capture**	1 s
**Stage temperature rate**	37°C
**Minimum cell size**	4 pixels
**Cell size**	13 pixels
**Minimum contrast**	30
**Cell intensity**	75 pixels
**Chamber type**	Slide-Coverslip (22*22 mm)
**Volume *per* slide**	7 μL
**Chamber depth**	≈ 20 μm
**Minimum number of field analysis**	500 cells
**Image type**	Phased contrast
**Average path velocity (VAP)**	50.0 μm/s
**Straightness (**	50%
**VAP cutoff**	10.0 μm/s
**Straight line velocity cutoff**	0.0 μm/s
**Curvilinear velocity**	>180 μm/s
**Amplitude of lateral head**	>9.5 μm
**Mean linearity**	<38%

#### 2.7.3 Morphology and viability of epididymal spermatozoa

Per WHO criteria [22], eosin-nigrosin staining evaluated morphology and viability at 400x magnification. Nonviable sperm exhibited a red stain, but viable sperm remained unstained. Two hundred spermatozoa per sample were examined for abnormalities, including deformities in the head, neck, and tail [21].

#### 2.7.4 Epididymal sperm DNA damage

Denatured DNA (red/yellow fluorescence) was isolated from native DNA by acridine orange labeling. Following fixation and staining with AO solution, the smears were examined under a 400x fluorescence microscope [21].

#### 2.7.5 Functionality of sperm plasma membrane (PMF)

In the hypo-osmotic swelling assay, 10 μL of sperm were incubated for 1 h at 37°C in 100 μL of hypo-osmotic solution (fructose 1.351 g/L, sodium citrate 0.735 g/L). Phase-contrast microscopy at 400x revealed coiled or inflated tails indicative of functional PMF [23].

### 2.8 Assessment of enzymatic antioxidant activity

Testicular tissues were homogenized in ice-cold lysis buffer (1:10 w/v) and centrifuged at 9000 rpm for 15 min at 4°C. Supernatants were collected for biochemical assays. Total antioxidant capacity (TAC), superoxide dismutase (SOD), and glutathione peroxidase (GPx) activities were determined using commercial colorimetric kits (Naxifer, Nagpix, and Nasdox, respectively; Navand Salamat, Iran). Lipid peroxidation was assessed by measuring malondialdehyde (MDA) levels with the Nalondi Lipid Peroxidation Assay Kit (Navand Salamat, Iran) at 523 nm. All procedures followed the manufacturers’ instructions. MDA concentrations were expressed as nmol per mg protein. Protein content was quantified using the Bradford method [21].

### 2.9 Histopathological and histomorphometric analysis of the testes

The right testis was fixed in Bouin's solution (preferred for better preservation of testicular architecture) or 10% neutral buffered formalin for 24–48 h, dehydrated through graded ethanol, cleared in xylene, and embedded in paraffin. Serial sections (5–7 μm thick) were prepared and stained with hematoxylin and eosin (H&E). Histological damage was evaluated using the Johnsen scoring system (scale 1–10) (Table 2) and Cosentino grading for I/R injury severity [24]. Mean seminiferous tubule diameter was measured in 100–200 round tubular cross-sections per testis using an ocular micrometer. Additional parameters including Sertoli cell index (SCI), repopulation index (RI), and meiotic index (MI) [25], tubular differentiation index (TDI) [26], Leydig cell nuclear diameter (LCND) [27], and spermiogenesis index (SPI) were also assessed [28]. All evaluations were performed by two independent observers blinded to the treatment groups under a light microscope (Olympus BH-2, Tokyo, Japan).

**Table 2:** Johnsen scoring system used for testicular damage evaluation.

Johnsen score	Description of histological criteria
10	Full spermatogenesis
9	Slightly impaired spermatogenesis, many late spermatids, disorganized epithelium
8	Less than five spermatozoa per tubule, few late spermatids
7	No spermatozoa, no late spermatids, many early spermatids
6	No spermatozoa, no late spermatids, few early spermatids
5	No spermatozoa or spermatids, many spermatocytes
4	No spermatozoa or spermatids, few spermatocytes
3	Spermatogonia only
2	No germinal cells, Sertoli cells only
1	No seminiferous epithelium

### 2.10 RNA extraction, cDNA synthesis, and real-time quantitative polymerase chain reaction (qRT-PCR)

Total RNA was extracted from testicular tissue using a standard TRIzol-based method or commercial RNA extraction kit. RNA quality and quantity were assessed by spectrophotometry (A260/A280 ratio). First-strand cDNA was synthesized from 1 to 2 μg total RNA using reverse transcriptase. Quantitative real-time PCR was performed on a StepOne Real-Time PCR System (Applied Biosystems, USA) with SinaSyber Blue HF-qPCR Mix (CinnaGen, Iran). Specific primers for Bax, Bcl-2, caspase-3, and the housekeeping gene GAPDH (glyceraldehyde-3-phosphate dehydrogenase) are listed in Table 3. The thermal cycling conditions consisted of initial denaturation at 95°C for 10 min, followed by 40–45 cycles of 95°C for 10 s, 60°C for 30 s, and 72°C for 30 s. Relative gene expression was calculated using the 2 ^(−ΔΔCt)^ method and reported as fold-change relative to the sham group [19].

**Table 3:** Nucleotide sequences and product size of primers used in reverse transcription-polymerase chain reaction.

Gene name	Primer	Band size
***Bcl-2***	Forward: 5- CTCGTCGCTACCGTCGTCACT TCG-3 Reverse: 5- CAGATGCCGGTTCAGGTACTCAGTC-3	242 bp
***Bax***	Forward: 5- ACC AGC TCTGAACAGATC ATG-3	424 bp
	Forward: 5- TGG TCT TGGATCCAGACAAG-3	
***caspase-3***	Forward: 5- GTACAGAGCTGGACTGCGGTATTG-3	84 bp
	Reverse: 5- AGTCGGCCTCCACTGGTATCTTC-3	
***GAPDH***	Forward: 5- TGGTGGACCTCATGGCCTAC - 3 Reverse: 5- CAGCAACTGAGGGCCTCTCT- 3	92 bp

### 2.11 Fertility index

On day 30 post-detorsion, each male mouse was co-housed with two virgin proven-fertile female mice (1:2 ratio) for a maximum of 72 h. Successful mating was confirmed by the presence of a vaginal plug (designated as gestational day 0). Females were maintained until gestational day 17, then euthanized under anesthesia. Uteri were examined for the number of implantation sites, viable fetuses, and litter size. Fertility rate (percentage of pregnant females) and mean litter size per pregnant female were recorded [29,30].

### 2.12 Statistical analysis

A one-way ANOVA was employed to analyze the differences among the Control, T/D, T/D + p-CA-50, T/D + p-CA-100, and T/D + p-CA-200 groups. The data were provided as mean ± standard deviation (SD). The Shapiro-Wilk and Levene's tests were employed to verify normality and homogeneity. Tukey's HSD post-hoc tests were conducted following significant ANOVA results. Utilizing SPSS 28.0, the significance threshold was *p* < 0.05 (IBM Corp., Armonk, NY, USA).

## 3 Results

### 3.1 Sperm quality parameters

Testicular torsion/detorsion (T/D) induced a substantial decline in epididymal sperm quality relative to the sham control group. Sperm concentration, total motility, progressive motility, and key kinematic parameters (VAP, VCL, VSL) were all significantly reduced (Table 4). Pretreatment with p-coumaric acid (p-CA) produced clear dose-dependent protection. The highest dose (200 mg/kg) yielded the most robust recovery, restoring most parameters to levels approaching those of the sham group. The 100 mg/kg dose also conferred substantial improvement, while the 50 mg/kg dose provided moderate but statistically significant benefit (p < 0.001 vs. T/D for 100 and 200 mg/kg groups). Linearity (LIN) and straightness (STR) remained unaffected across all groups (p > 0.05).

**Table 4:** Epididymal sperm concentration, total and progressive motilities, and motility characteristics in different experimental groups. Values are expressed as mean ± SD.

Analysis	Control	T/D	T/D + p-CA-50	T/D + p-CA-100	T/D + p-CA-200
**Epididymal sperm concentration (10^6^/mL)**	32.45 ± 2.78^a^	16.12 ± 3.45^e^	24.12 ± 2.94 ^d^	28.89 ± 2.31^c^	30.92 ± 2.41^b^
**Total motility (%)**	80.23 ± 1.92^a^	45.67 ± 2.56 ^d^	63.45 ± 1.83^c^	75.07 ± 1.46^b^	78.14 ± 3.08 ^ab^
**Progressive motility (%)**	41.06 ± 1.91^a^	8.32 ± 1.89 ^d^	29.67 ± 1.21^c^	34.83 ± 1.02^b^	38.95 ± 2.45^a^
**VAP (μm/s)**	29.62 ± 1.34^a^	14.78 ± 1.98 ^d^	18.94 ± 1.36^c^	25.36 ± 1.20^b^	27.89 ± 1.75 ^ab^
**VCL (μm/s)**	84.12 ± 2.21^a^	42.89 ± 2.67 ^d^	68.76 ± 1.53^c^	78.58 ± 1.26^b^	81.94 ± 1.55 ^ab^
**VSL (μm/s)**	23.68 ± 1.29^a^	11.15 ± 1.94 ^d^	16.34 ± 1.94^c^	20.89 ± 1.05^b^	22.47 ± 1.65 ^ab^
**LIN (%)**	0.24 ± 0.03^a^	0.21 ± 0.04^a^	0.22 ± 0.02^a^	0.22 ± 0.01^a^	0.22 ± 0.03^a^
**ALH (μm/s)**	2.73 ± 0.02^a^	1.09 ± 0.03 ^d^	1.89 ± 0.02^c^	2.49 ± 0.03^b^	2.67 ± 0.01 ^ab^
**STR (%)**	0.86 ± 0.02^a^	0.83 ± 0.04^a^	0.83 ± 0.03^a^	0.84 ± 0.02^a^	0.85 ± 0.03^a^
**BCF (Hz)**	12.79 ± 1.12^a^	6.32 ± 1.45 ^d^	8.76 ± 1.25^c^	11.68 ± 1.10^b^	12.34 ± 1.19 ^ab^

- T/D: Torsion/detorsion; T/D + p-CA-50: T/D + 50 mg/kg p-coumaric acid, etc. Different superscripts within a row indicate significant differences (p ≤ 0.05). VAP: average path velocity; VCL: curvilinear velocity; VSL: straight-line velocity; LIN: linearity; ALH: amplitude of lateral head displacement; STR: straightness; BCF: beat-cross frequency.

### 3.2 Sperm functionality, viability, and morphology

T/D significantly increased the percentage of spermatozoa with damaged DNA (acridine orange assay), abnormal morphology, and compromised plasma membrane integrity while decreasing viability (Table 5). p-CA treatment markedly attenuated these deficits in a dose-dependent fashion. The 200 mg/kg dose provided the strongest protection, reducing DNA fragmentation and morphological abnormalities to near-control levels and improving membrane functionality and viability. The 100 mg/kg dose showed comparable efficacy to the 200 mg/kg dose for several parameters, whereas the 50 mg/kg dose produced moderate yet significant recovery (p < 0.01–0.001).

**Table 5:** Epididymal sperm plasma membrane functionality, DNA damage, viability and abnormal morphology in different experimental groups. Values are expressed as mean ± SD.

Analysis	Control	T/D	T/D + p-CA-50	T/D + p-CA-100	T/D + p-CA-200
Sperm plasma membrane functionality (%)	83.17 ± 2.45^a^	48.29 ± 3.92 ^d^	66.03 ± 2.10^c^	77.89 ± 3.75^b^	80.94 ± 3.69 ^ab^
Sperm viability (%)	87.03 ± 2.19^a^	55.28 ± 3.67 ^d^	71.89 ± 2.91^c^	82.42 ± 2.13^b^	85.47 ± 3.46 ^ab^
Sperm DNA damage (%)	5.08 ± 0.20^e^	29.14 ± 1.63^a^	15.12 ± 1.24^b^	9.36 ± 0.62^c^	7.29 ± 0.59 ^d^
Sperm abnormal morphology (%)	7.19 ± 0.39^e^	34.92 ± 1.48^a^	18.21 ± 1.07^b^	11.93 ± 0.51^c^	9.87 ± 0.53 ^d^

- T/D: Torsion/detorsion; T/D + p-CA-50: T/D + 50 mg/kg p-coumaric acid, etc. Different superscripts within a row indicate significant differences (p ≤ 0.05).

### 3.3 Histological parameters and reproductive organ weights

**Fig. 1:**
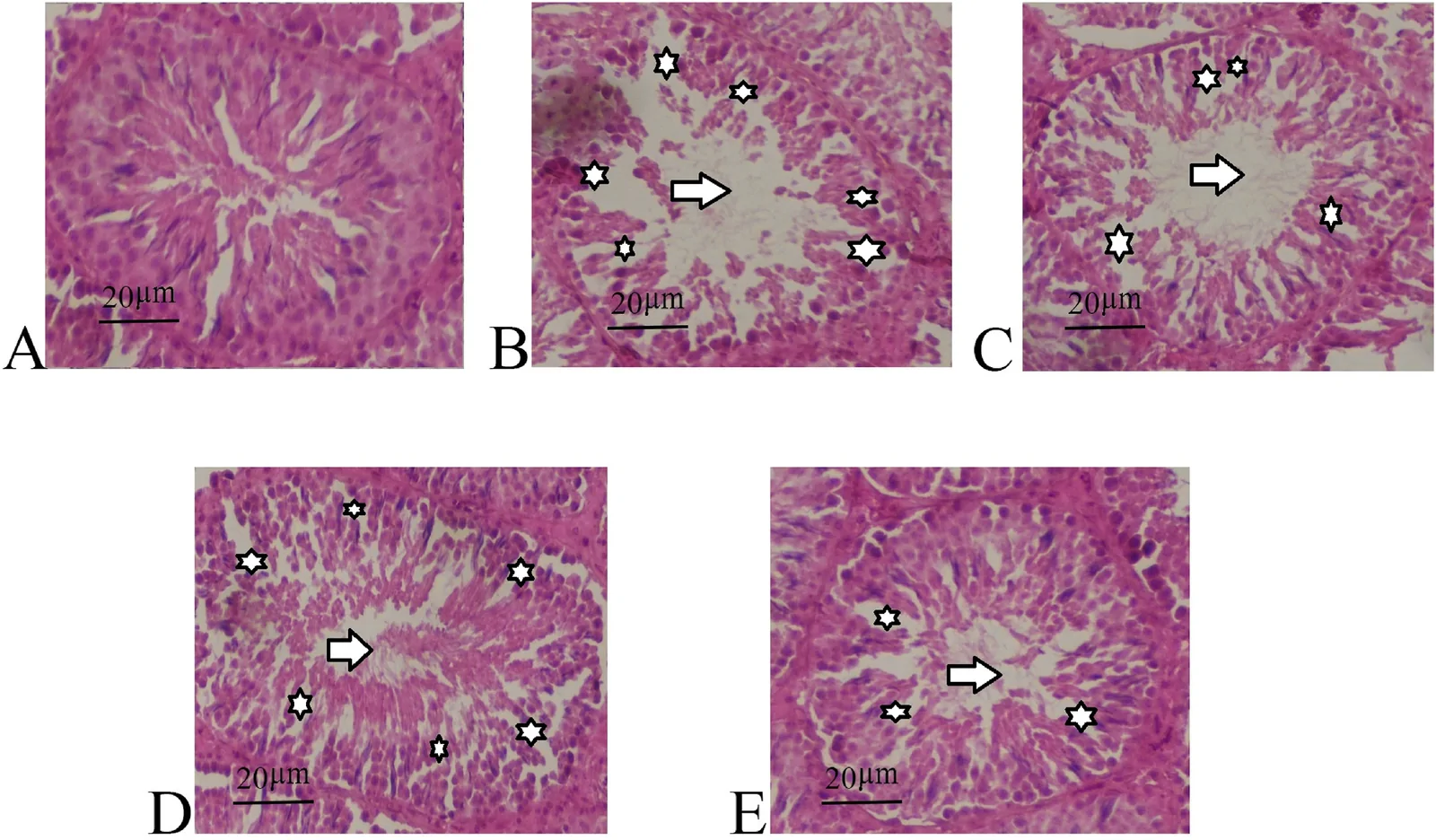
Representative photomicrographs of testicular histo-architecture in different experimental groups (H&E staining, 400× magnification). (A) Sham control showing normal seminiferous tubule architecture with full spermatogenesis. (B) Torsion/detorsion (T/D) group exhibiting severe germ cell sloughing, vacuolization, tubular atrophy, and interstitial edema. (C–E) T/D + p-CA at 50, 100, and 200 mg/kg, respectively, demonstrating dose-dependent preservation of tubular integrity and spermatogenic activity. White arrows indicate sperm in the tubular lumen; asterisks highlight areas of disorganization. Scale bar = 20 μm.

Body weights did not differ significantly among groups at the end of the study (Table 6). In contrast, T/D caused marked reductions in testicular weight, volume, and testis-to-body weight ratio, consistent with atrophy (Fig. 1, Table 6). p-CA pretreatment preserved organ weights in a dose-dependent manner, with the 200 mg/kg dose yielding values closest to sham controls.

**Table 6:** Histological parameters and reproductive organ weights in different experimental groups. Values are expressed as mean ± SD.

Analysis	Groups				
	Control	T/D	T/D + p-CA-50	T/D + p-CA-100	T/D + p-CA-200
**length of the right testis (cm)**	0.79 ± 0.02^a^	0.63 ± 0.04 ^d^	0.69 ± 0.03^c^	0.74 ± 0.04^b^	0.77 ± 0.03^a^
**length of the left testis (cm)**	0.80 ± 0.02^a^	0.66 ± 0.04 ^d^	0.71 ± 0.02^c^	0.75 ± 0.03^b^	0.78 ± 0.02 ^ab^
**Diameter of the right testis (cm)**	0.50 ± 0.02^a^	0.37 ± 0.03 ^d^	0.42 ± 0.01^c^	0.46 ± 0.02^b^	0.48 ± 0.03 ^ab^
**Diameter of the left testis (cm)**	0.50 ± 0.03^a^	0.35 ± 0.03 ^d^	0.41 ± 0.03^c^	0.44 ± 0.03^b^	0.48 ± 0.02^a^
**1**st**-day body weight (g)**	25.34 ± 0.03^a^	25.12 ± 0.04^a^	25.46 ± 0.02^a^	25.30 ± 0.02^a^	25.27 ± 0.03^a^
**35-day body weight (g)**	26.52 ± 0.02^a^	18.47 ± 0.04^c^	21.92 ± 0.02^b^	24.65 ± 0.03^a^	25.78 ± 0.03^a^
**Right testis weight (g)**	0.113 ± 0.002^a^	0.066 ± 0.003 ^d^	0.094 ± 0.002^c^	0.107 ± 0.004^b^	0.110 ± 0.001^a^
**Left Testis weight (g)**	0.111 ± 0.001^a^	0.066 ± 0.001 ^d^	0.091 ± 0.003^c^	0.106 ± 0.002^b^	0.109 ± 0.003^a^
**Epididymis weight (g)**	0.044 ± 0.002^a^	0.027 ± 0.002 ^d^	0.033 ± 0.002^c^	0.037 ± 0.003^b^	0.042 ± 0.001^a^
**Testis/body weight (%)**	0.43 ± 0.001^a^	0.25 ± 0.004 ^d^	0.35 ± 0.002^c^	0.39 ± 0.002^b^	0.41 ± 0.001 ^ab^
**Johnsen score**	9.45 ± 0.19^a^	5.48 ± 0.25 ^d^	7.15 ± 0.21^c^	8.51 ± 0.15^b^	9.03 ± 0.12 ^ab^
**Cosentino score**	1.01 ± 0.02^c^	3.41 ± 0.04^a^	2.34 ± 0.03^b^	1.25 ± 0.02^c^	1.15 ± 0.02^c^
**Seminiferous tubule diameter (μm)**	54.62 ± 1.15^a^	31.05 ± 1.38^e^	38.12 ± 1.17 ^d^	46.97 ± 1.10^c^	51.84 ± 1.02^b^
**Sertoli cell index**	85.56 ± 2.45^a^	69.47 ± 2.58 ^d^	74.03 ± 2.08^c^	81.94 ± 2.64^b^	83.92 ± 1.84 ^ab^
**Repopulation index**	70.78 ± 2.56^a^	43.92 ± 1.25 ^d^	56.12 ± 1.29^c^	65.22 ± 1.53^b^	68.45 ± 1.59 ^ab^
**Miotic index**	2.71 ± 0.02^a^	1.06 ± 0.05 ^d^	1.79 ± 0.02^c^	2.44 ± 0.03^b^	2.62 ± 0.03^a^
**Leydig cell nuclear diameter (μm)**	6.15 ± 0.19^c^	8.16 ± 0.23^a^	7.20 ± 0.16^b^	6.47 ± 0.19^c^	6.29 ± 0.11^c^
**Tubular differentiation index (%)**	86.03 ± 1.89^a^	70.08 ± 2.36 ^d^	75.92 ± 2.29^c^	82.12 ± 1.53^b^	84.56 ± 1.98 ^ab^
**Spermiogenesis index (%)**	83.12 ± 1.48^a^	56.45 ± 1.39 ^d^	70.98 ± 1.60^c^	78.29 ± 1.24^b^	81.34 ± 1.69 ^ab^

- T/D: Torsion/detorsion; T/D + p-CA-50: T/D + 50 mg/kg p-coumaric acid, etc. Different superscripts within a row indicate significant differences (p ≤ 0.05).

Histological examination revealed severe damage in the T/D group, including germ cell sloughing, vacuolization, seminiferous tubule atrophy, interstitial edema, and inflammatory infiltration. Johnsen scores were significantly lower and Cosentino grades higher compared with sham (Table 6). p-CA treatment dose-dependently ameliorated these changes. The 200 mg/kg group exhibited near-normal tubular architecture and cellular organization, while the 100 mg/kg group showed substantial improvement and the 50 mg/kg group provided moderate protection. Seminiferous tubule diameter and quantitative indices of spermatogenesis (SCI, RI, TDI, SPI, MI) followed the same pattern of dose-dependent recovery (p < 0.001 for 100 and 200 mg/kg vs. T/D).

### 3.4 Fertility outcomes

T/D markedly impaired male fertility, resulting in significantly lower pregnancy rates and litter sizes compared with the sham group (Table 7). p-CA pretreatment improved fertility indices dose-dependently. The 200 mg/kg dose restored pregnancy rate and litter size most effectively, approaching sham levels, followed by the 100 mg/kg dose. The 50 mg/kg dose produced a moderate but significant improvement (p < 0.01). Mating behavior (plug formation) remained unaffected across groups.

**Table 7:** Fertility indexes of adult male mice following testicular torsion/detorsion (T/D) and p-coumaric acid (p-CA) pretreatment, after natural mating with non-exposed females.

Analysis	Control	T/D	T/D + p-CA-50	T/D + p-CA-100	T/D + p-CA-200
**Number of females**	20	20	20	20	20
**Number of females mated**	20	20	20	20	20
**Number of males mated**	10	10	10	10	10
**Number of males impregnating females**	10	1	4	6	9
**Number of females pregnant**	20	1	5	8	16
**Female mating index (%)**	100^a^	100^a^	100^a^	100^a^	100^a^
**Male mating index (%)**	100^a^	100^a^	100^a^	100^a^	100^a^
**Pregnancy index (%)**	100^a^	5 ^d^	25^c^	40^c^	80^b^
**Male fertility index (%)**	100^a^	10 ^d^	40^c^	60^b^	90^a^

- T/D: Torsion/detorsion; T/D + p-CA-50: T/D + 50 mg/kg p-coumaric acid, etc. Different superscripts within a row indicate significant differences (p ≤ 0.05). Female mating index = number of females mated/number of females × 100; Male mating index = number of males mated/number of males × 100; Pregnancy index = number of females pregnant/number of females mated × 100; Male fertility index = number of males impregnating females/number of males mated × 100.

### 3.5 Oxidative stress markers, hormone levels, and apoptotic gene expression

**Fig. 2:**
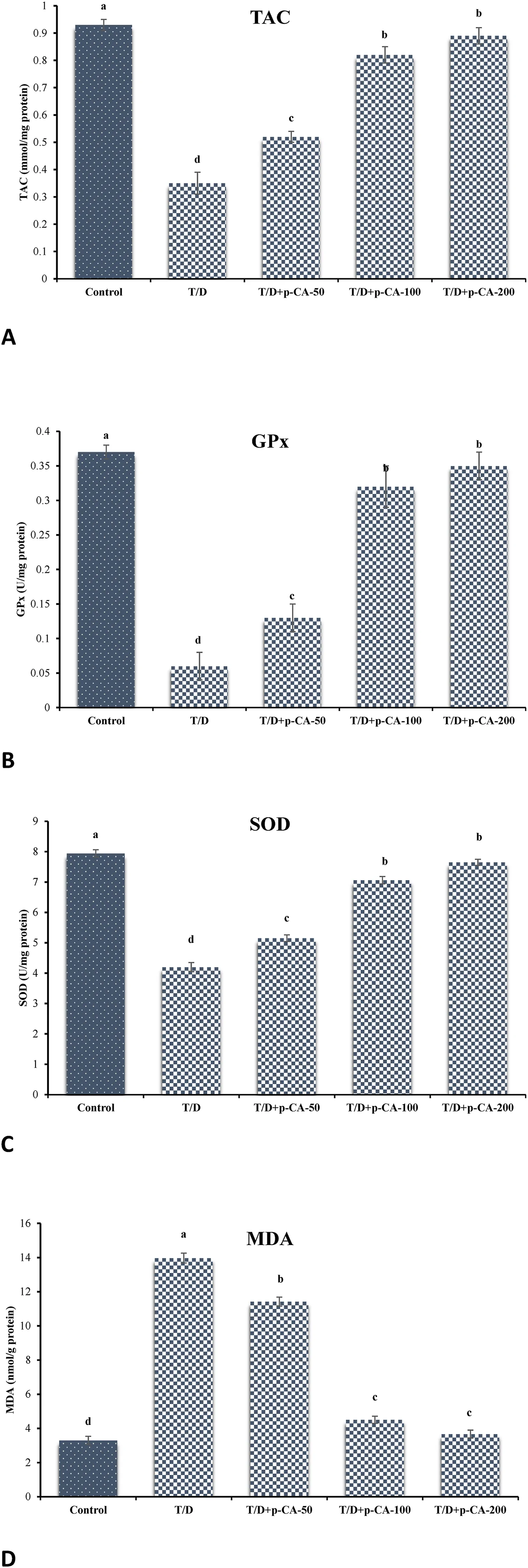
Effects of p-CA on testicular oxidative stress markers. (A) Total antioxidant capacity (TAC), (B) Glutathione peroxidase (GPx), (C) Superoxide dismutase (SOD), and (D) Malondialdehyde (MDA) levels in different groups. Bars represent mean ± SD. Different superscripts indicate significant differences (p ≤ 0.05). T/D: torsion/detorsion; p-CA: p-coumaric acid.

**Fig. 3:**
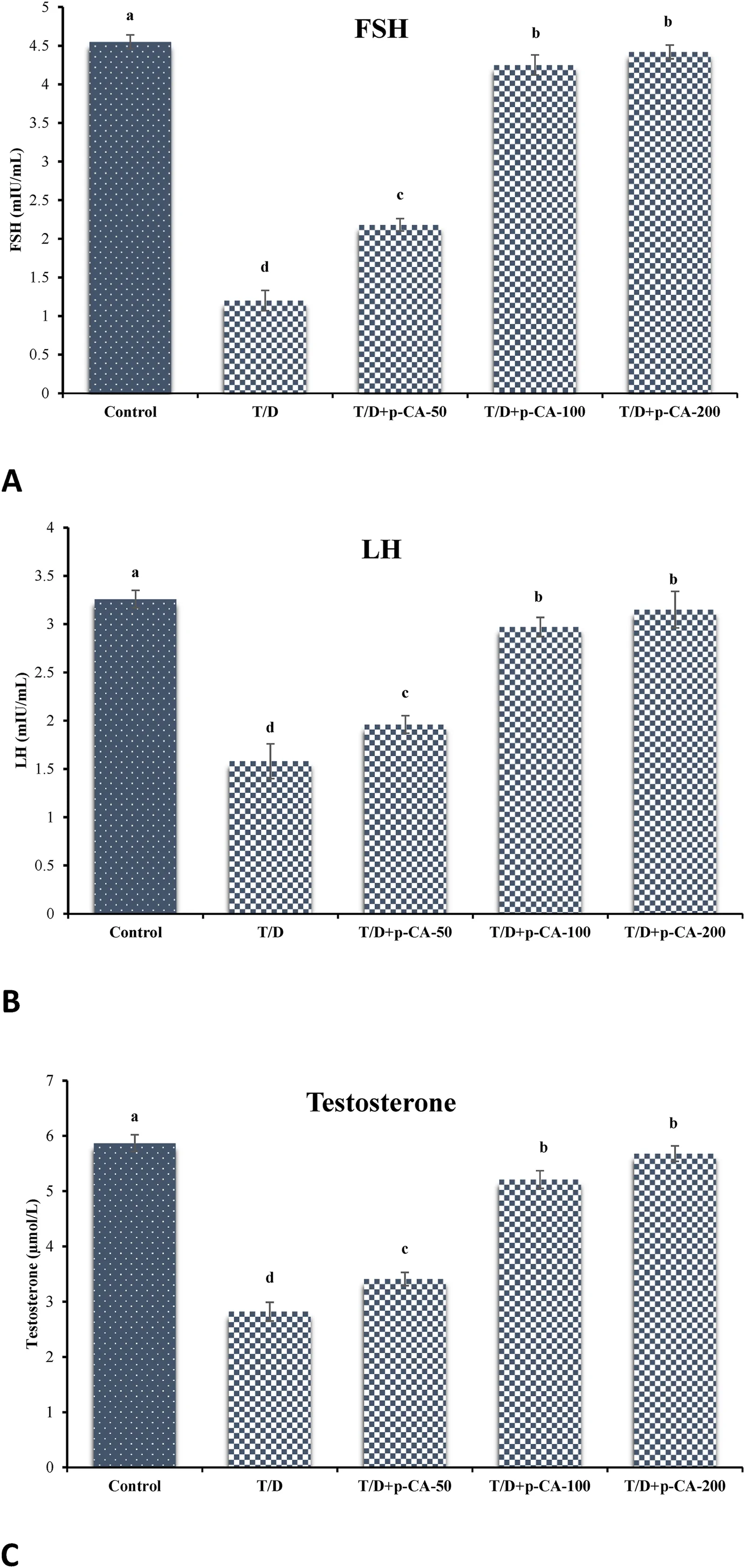
Serum hormone concentrations. (A) Follicle-stimulating hormone (FSH), (B) Luteinizing hormone (LH), and (C) Testosterone levels across experimental groups. Bars represent mean ± SD. Different superscripts denote significant differences (p ≤ 0.05).

**Fig. 4:**
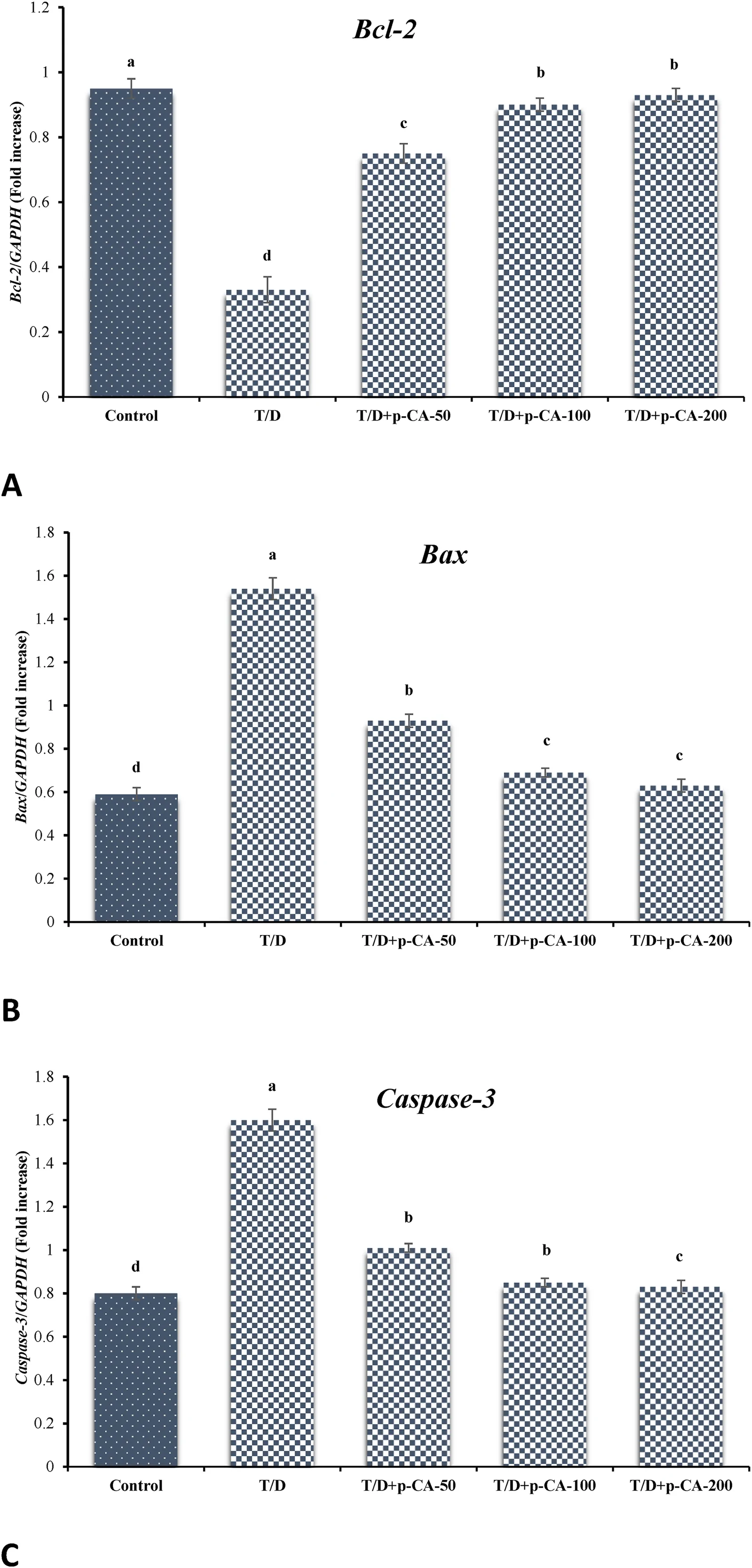
Relative mRNA expression of apoptotic genes in testicular tissue. (A) Bcl-2, (B) Bax, and (C) Caspase-3. Expression levels were normalized to GAPDH using the 2 ^(−ΔΔCt)^ method. Bars represent mean ± SD. Different superscripts indicate significant differences (p ≤ 0.05).

T/D significantly depleted antioxidant defenses (↓ TAC, SOD, GPx) and elevated lipid peroxidation (↑ MDA) (Fig. 2). Concurrently, circulating testosterone, LH, and FSH levels were suppressed (Fig. 3). At the molecular level, T/D upregulated pro-apoptotic Bax and caspase-3 while downregulating anti-apoptotic Bcl-2 (Fig. 4).

p-CA pretreatment restored redox balance, hormone levels, and apoptotic gene expression in a clear dose-dependent manner. The 200 mg/kg dose produced the strongest normalization, often approaching sham values, followed by 100 mg/kg. The 50 mg/kg dose offered moderate but significant protection across all parameters (p < 0.01–0.001 for 100 and 200 mg/kg groups vs. T/D).

## 4 Discussion

Spermatic cord torsion in pediatric patients constitutes a critical urological emergency that may lead to infertility if not promptly addressed. Testicular atrophy and degeneration occur due to twisting, which interrupts the blood supply [31]. Testicular tissue is more susceptible to hypoxia and free radicals due to its elevated rate of cellular proliferation and oxygen-utilizing spermatogenesis [31]. The principal pathologic consequence of delayed detorsion is the demise of germ cells resulting from inadequate reperfusion [32]. Male infertility correlates with increased ROS levels, which adversely affect testicular cells and spermatozoa [33]. Omeprazole, hesperidin, and diclofenac have demonstrated protective effects against ischemia/reperfusion injury in testes [[34], [35], [36], [37], [38], [39], [40], [41]]. This study is, to our knowledge, the first to examine the dose-dependent efficacy of intraperitoneal p-CA in mitigating testicular ischemia/reperfusion injury in a mouse model, and the first to link this protection to a coherent chain of evidence spanning molecular apoptotic regulation, tissue-level histomorphometry, sperm functional competence, and actual fertility outcomes. The results indicated that p-CA mitigated T/D-induced testicular tissue damage and apoptosis, enhanced sperm quantity and quality, and restored the oxidant-antioxidant equilibrium.

Numerous investigations utilizing animal models have examined the function of antioxidants, including phytochemicals and enzymes, in mitigating testicular T/D harm [42]. For instance, in T/D models, lycopene promotes motility and decreases faulty sperm, whereas melatonin decreases sperm abnormalities [43]. Following I/R, ghrelin enhances sperm concentration and motility [44]. Consistent with prior research, our findings indicate that 720° T/D adversely affects spermatogenesis in mice by markedly reducing sperm count, progressive motility, and kinematic parameters [45]. Mechanisms like Ca^2+^ signaling and cAMP-dependent protein kinase A are critical for progressive motility [21,[46], [47], [48]]. Mitochondria, dependent on oxidative phosphorylation for ATP production, are vulnerable to T/D, which can disrupt energy metabolism and motility [49,50]. Our results demonstrate that intraperitoneal p-CA at doses of 50, 100, and 200 mg/kg increases sperm motility, concentration, and membrane integrity in a dose-dependent manner while decreasing DNA damage and abnormal morphology — effects that compare favorably with or surpass those reported for naringin at similar doses in previous studies.

In testicular tissue, semen, and spermatozoa, antioxidant enzyme activity (SOD, CAT, GPx) and the lipid peroxidation marker MDA together reflect oxidative status; the enzymes mitigate damage inflicted by free radicals. Nonetheless, these enzymes are overwhelmed by excessive reactive oxygen species during ischemia/reperfusion [51]. Our analysis demonstrated that 720° T/D decreased TAC capacity and GPx/SOD activity while elevating MDA levels. The injection of p-CA enhanced antioxidant defenses. This is consistent with previous reports showing that p-CA reduces MDA and enhances SOD/GPx activity in renal and other I/R models [52]**.** The bioactive constituents of p-CA safeguard against oxidative stress induced by reactive oxygen species [53].

Reperfusion can lead to tissue injury and elevation in apoptosis [54]. Testicular I/R utilizes ROS generated by neutrophils to induce germ cell death [55]. Apoptosis is executed by caspase-3, resulting in chromatin condensation and DNA fragmentation [56]. Our research indicates that p-CA following T/D decreased apoptosis in the testes of mice by elevating Bcl-2 expression and reducing Bax and caspase-3. This aligns with earlier findings on p-CA in other oxidative stress models [57].

Infertility risk escalates when testicular torsion disrupts the blood-testis barrier [58]. Prior studies demonstrate that diminished serum testosterone and sperm quality following T/D lead to a reduction in pregnancies and embryos [59]. Our investigation demonstrated that p-CA boosted fertility through improved sperm motility, viability, and membrane integrity.

## 5 Limitations

While mice models facilitate genetic modification, their clinical relevance may be constrained. The study employed three intraperitoneal doses of p-CA, potentially overlooking optimal ranges or other administration routes. The 30-day timeframe limited very long-term assessments. Systemic effects and detailed pharmacokinetics of p-CA were not evaluated. Administering p-CA 30 min before detorsion assumes advance knowledge of the timing of surgical intervention, which may not reflect the often unpredictable and delayed clinical presentation of testicular torsion; future studies should therefore also evaluate p-CA efficacy when administered at, or shortly after, detorsion to better inform clinical applicability. The doses selected (50-200 mg/kg) were based on prior rodent studies in which comparable or higher doses of p-CA were administered without reports of overt systemic toxicity; nonetheless, formal toxicological evaluation of these doses was beyond the scope of the present study and should be addressed before any clinical translation is considered.

## 6 Conclusion

At doses of 100 and 200 mg/kg, p-coumaric acid effectively mitigates testicular ischemia/reperfusion injury in mice by elevating antioxidant levels, enhancing sperm quality, boosting fertility, and reducing oxidative stress and apoptosis. By connecting molecular, histological, functional, and reproductive endpoints within a single dose-response design, this study provides the first integrated evidence for p-CA as a candidate perioperative adjunct in the clinical management of testicular torsion, and identifies 100–200 mg/kg as the dose range warranting further translational investigation.

## CRediT authorship contribution statement

**Khashayar Khorsand:** Conceptualization, Data curation, Methodology, Resources, Software, Supervision, Visualization, Writing – original draft, Writing – review & editing. **Alireza Najafpour:** Conceptualization, Data curation, Formal analysis, Funding acquisition, Investigation, Methodology, Project administration, Resources, Software, Supervision, Validation, Visualization, Writing – original draft, Writing – review & editing. **Mohammadreza Valilou:** Conceptualization, Data curation, Formal analysis, Methodology, Project administration, Resources, Software, Supervision, Writing – original draft, Writing – review & editing. **Ali Soleimanzadeh:** Conceptualization, Data curation, Investigation, Resources, Validation, Visualization, Writing – original draft, Writing – review & editing.

## Ethics declaration

This study was conducted in accordance with the ARRIVE (Animal Research: Reporting of In Vivo Experiments) guidelines. This study was approved by the Islamic Azad University. (Approval No. IR.IAU.URMIA.REC.1403.092)

## Ethics approval and consent to participate

All of the protocols were approved by the Faculty of Veterinary Medicine's Committee on the Ethics of Animal Experiments at Islamic Azad University. Every procedure was carried out in accordance with the relevant laws and standards. The study was conducted in compliance with the ARRIVE standards. The owner(s) of the animals gave their informed consent for us to use them in the study. The research adhered to the guidelines established by the Islamic Azad University Animal Ethics Committee (IR.IAU.URMIA.REC.1403.092).

## Declaration of generative AI in scientific writing

During the preparation of this work the author(s) used ChatGPT to improve the clarity and readability of the text. After using this tool/service, the author(s) reviewed and edited the content as needed and take(s) full responsibility for the content of the publication.

## Funding

This research did not receive any specific grant from funding agencies in the public, commercial, or not-for-profit sectors.

## Declaration of competing interest

The authors declare that they have no known competing financial interests or personal relationships that could have appeared to influence the work reported in this paper.

## Data Availability

Data will be made available on request.
